# The effect of pueraria lobata/rehmannia glutinosa and exercise on fatty acid transporters expression in ovariectomized rats skeletal muscles

**DOI:** 10.20463/jenb.2016.09.20.3.5

**Published:** 2016-09-30

**Authors:** Hye Jin Kim, Hae Min Yoon, Oran Kwon, Won Jun Lee

**Affiliations:** 1 Department of Kinesiology and Sports Studies, College of Science and Industry Convergence, Ewha Womans University, Seoul Republic of Korea; 2 Department of Nutritional Science and Food Management, College of Science and Industry Convergence, Ewha Womans University, Seoul Republic of Korea

**Keywords:** Menopause, FATP1, FABPpm, FAT/CD36, Pueraria lobata/Rehmannia glutinosa, aerobic exercisetion

## Abstract

**[Purpose]:**

Pueraria lobata/rehmannia glutinosa (PR) and exercise have been receiving a lot of attention from postmenopausal women, as a result of the side effects of estrogen replacement therapy. However, the effects of PR and exercise on fatty acid transporters (FATPs), which play essential role in fatty acid transport, have not been studied. In this study, we evaluated the effects of PR and aerobic exercise on FATP1, FABPpm and FAT/CD36 expression in ovariectomized rat skeletal muscles.

**[Methods]:**

Sixty rats were randomly divided into 6 groups: (1)HSV; high fat diet (HFD)+sedentary+vehicle, (2)HSP; HFD+sedentary+PR, (3)HSH; HFD+sedentary+17β-estradiol, (4)HEV; HFD+exercise+vehicle, (5) HEP; HFD+exercise+PR, (6)HEH; HFD+exercise+17β-estradiol. Exercise consisted of treadmill exercise (1-4th week: 15 m/min for 30 min, 5-8th week: 18 m/min for 40 min, 5 times/week).

**[Results]:**

Exercise does not alter FATP1 and FAT/CD36 gene levels in soleus and plantaris muscles. In contrast, exercise had main effect on up-regulation of FABPpm mRNA expression in both muscles. However, FABPpm level was not increased by exercise combined with treatments, indicative of no additive effects of PR or hormone on FABPpm gene expression. On the other hand, immunohistochemistry result showed that translocation of FATPs proteins to plasma membrane were higher in PR, exercise groups, and exercise combined with PR groups in both muscles.

**[Conclusion]:**

These result showed that aerobic exercise and PR may help increase fat-oxidation through the induction of FABPpm, a muscle specific transporter, in OVX rat skeletal muscles. In addition, FABPpm expression is possibly regulated post-transcriptionally in exercise, or pre-translationally in PR.

## INTRODUCTION

Overweight and obesity are critical social problems, because they are associated with major causes of various diseases such as cardiovascular disease, type 2 diabetes mellitus, hypertension and so on ^1^^,^ ^2^. Especially, many postmenopausal women are overweight or obese and are exposed to the risk of related diseases from hormonal changes during the menopausal period^3^. During the menopausal years, the level of estrogens is decreased significantly, and that affects women’s life in several ways. The loss of estrogens is the main reasons of bone loss and osteoporosis in women after menopause period^4^. In addition, estrogen deficiency could directly mediate imbalance of lipid metabolism in postmenopausal women^5^. Previous studies have shown that fat mass and the waist-to-hip ratio were greatly increased, but loss of fat-free mass was significant in postmenopausal women^6^^,^ ^7^. In addition, many postmenopausal women suffer from the metabolic syndromes, which are abdominal adiposity, insulin resistance, and dyslipidemia caused by estrogen deficiency^8^.

Estrogen replacement therapy (ERT) is one of the common ways to improve the symptoms of estrogen deficiency. ERT influences positively on overall postmenopausal women’s life by declining the risk of cardiovascular disease, osteoporosis, stroke, and also Alzheimer’s disease^9^^,^ ^10^. However, recent studies have shown the side effects of ERT such as vaginal bleeding, thrombosis, hypertension, and so on^9^. Because of these side effects of ERT, natural resources such as pueraria lobata and rehmannia glutinosa, which have the efficacy of estrogen, have received much attention.

Pueraria lobata is a well-known herb in Asian countries and contains abundant isoflavones called puerarian. Isofalvone are used to manage several age-related chronic disease such as diabetes, atherosclerosis, cardiovascular diseases, and inflammation^11^. Especially, puerarin contains abundant phytoestrogens, which are natural hormone-like compounds and work as estrogens that can improve the symptoms of estrogen deficiency such as dyslipidemia and osteoporosis^12^. Rehmannia glutinosa is another widely used herbal medicine, which is commonly used in combination with pueraria lobata. Catalpol, which is contained in rehmannia glutinosa, has beneficial treatment effects on anti-inflammation, reduction of blood glucose, protection of liver damage, and alimentation of diabetes^13^. Catalpol is also helpful to menopausal women by increasing the production of sex hormones and improving the balance of estrogen, follicle stimulating hormone (FSH) and luteinizing hormone (LH)^13^^,^ ^14^. However, the combination of puerarin and catalpol has not been well studied.

Exercise is another non-pharmacological treatment to lower the symptoms of postmenopausal women caused by estrogen deficiency. Exercise is known to have tremendous beneficial effects on health. Besides, the influences of exercise among the postmenopausal women are not exceptions. Exercise is related to enhancement of self-esteem and self-efficacy, which helps the improvement of depression and other psychological problems^15^. In addition, exercise can alleviate distress of physical problems, for example, hot flush, fatigue, sleep disorders, and muscle-joint problems in postmenopausal women^16^.

In postmenopausal women, obesity and type 2 diabetic patients, the triacylglycerol (TG) content in rectus abdominis muscle is largely increased, as compared with normal people; and high level of intramyocellular triacylglycerol (IMTG) accumulation has negative correlation with insulin sensitivity^17^^-^^19^. Long-chain fatty acid (LCFA) is a major energy source of cell metabolism and plays an important role in the synthesis of TG, which is used for storage of metabolic energy^20^. However, the increase of LCFA uptake and TG synthesis/storage, and the decrease of LCFA oxidation in skeletal muscles lead to insulin resistance that results in obesity and diabetes, as well as metabolic syndrome and cardiovascular disease^18^^,^ ^21^.

Maintaining the proper balance between LCFA uptake and oxidation is a crucial mechanism for prevention or improvement of various metabolism-related diseases. Fatty acid transporters play central roles in maintaining the balance between LCFA uptake and oxidation. Fatty acid transporters mediate lipid metabolism by LCFA transport in diverse organizations, as well as in skeletal muscles. FATP1 (fatty acid transporter 1), FABPpm (plasma membrane-bound fatty acid binding protein), and FAT/CD36 (fatty acid translocase) are the three major fatty acid transporter proteins^22^.

As a result, for the postmenopausal women, the balance of lipid metabolism is essential to prevent obesity, metabolic syndrome, and cardiovascular diseases. ERT, natural resources and exercise have beneficial effect on decreasing fat mass in postmenopausal women. However, the mechanisms involved in fat mass are unclear. To better understand the effect of pueraria lobata/rehmannia glutinosa (PR), and exercise on lipid metabolism, it is necessary to determine the change of expression of fatty acid transporters by PR, and exercise. Therefore, the purpose of this study was to determine the effects of PR, and aerobic exercise on the expression of fatty acid transporters in ovariectomized (OVX) rats skeletal muscle.

## METHODS

### Human and animal rights and informed consent

All animal experiments were approved by the Institutional Animal Care and Use Committee (IACUC) of EWHA Womans University, Seoul, Korea. Permit Number: 14-038.

### Animal care and treatment

**Figure 1.**
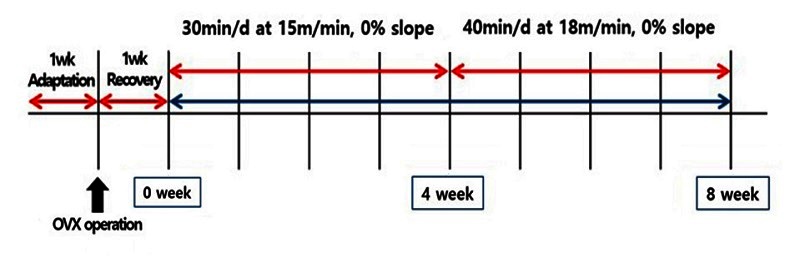
Experimental design and exercise protocol

Sixty female Sprague-Dawley (SD) rats (206.19 ± 6.2 g), aged 8weeks, were obtained from Central Lab Animal (Seoul, Korea). Animals were housed in an air-conditioned room at 23 ± 1℃, 64. 1 % relative humidity and a 12 h light/dark cycle. The experimental diets were purchased from Research Diet Inc. (New Brunswick, NJ, USA): the high fat diet contained 45 % of energy as fat from corn oil plus lard (225:1598) (Product# D12451). Ovaries of all rats were removed surgically to induce postmenopausal status and 1 week of recovery period was given. After recovery periods, rats were randomly divided into 6 groups following treatment groups: (1) high fat diet (HFD) + sedentary + vehicle (HSV), (2) HFD + sedentary + PR (complex of pueraria lobata/rehmannia glutinosa by 3 to 1; 400 mg/kg body weight/day) (HSP), (3) HFD + sedentary + 17β-estradiol (0.5 mg/kg body weight/day) (HSH), (4) HFD + exercise + vehicle (HEV), (5) HFD + exercise + PR (HEP), (6) HFD + exercise + 17β-estradiol (HEH). After 1 week of rest period, the animals were acclimated to running on a treadmill for 15 min, 8 m/min, with 0° of inclination for one day. Subsequently, the animals were regularly exercised 5 times per week for 8 weeks and the running started at 10 o’clock in the morning. From 1st week to 4th week, the animals ran on a treadmill form 30 min/day, and 15 m/min with 0° of inclination. Subsequently, from 5th week to 8th week, the running progressed for 40 min/day and 18 m/min with 0° of inclination (Fig. 1).

### RNA extract and quantitative real-time PCR

Total RNA was extracted from soleus and plantaris muscles using a TRIzol reagent (Invitrogen, Carlsbad, CA, USA). The RNA concentration and quality were measured at 260/280 nm using a spectrophotometer (Nanodrop-2000, Thermo Fisher Scientific, Waltham, MA). Next, cDNA was synthesized from 1 μg of total RNA in the presence of random primer, 2.5 mM dNTP, RNase inhibitor, and reverse transcriptase (Invitrogen Life Technologies, Carlsbad, CA) in a final volume of 20 μl at 25℃ for 10 min, followed by 42℃ for 60 min and 95℃ for 5 min. Real-time qPCR was performed in Step-One-Plus Real time-PCR System (Applied Biosystems, Foster City, CA, USA). PCR was performed in duplicate using the SYBR Green PCR master mix (Kappa, USA) according to the manufacturer’s instructions. The primer sets for rat target genes were showed in Table1. The primers were purchased from Macrogen (Macrogen, KOREA). The expression of the target genes was then normalized to the expression of glyceraldehyde 3-phosphate dehydrogenase (GAPDH). All data were expressed as a relative quantity to each control value.

**Table 1:** Primer sequences for real-time qPCR

Gene	Forward primers (5’-> 3’)	Reverse primers (5’-> 3’)
GAPDH	5’-TGCACCACCAACTGCTTA-3’	5’-GGATGCAGGGATGATGTTC-3’
FATP1	5’-GCAAGCCAGAGAAGGATGCG-3’	5’-CCAAAGAGGTCTCGCCTCG-3’
FABPpm	5’-GGAGGGTGGATGGTGTTGAG-3’	5’-TCCAGATATCAGCCGTGGGA-3’
FAT/CD36	5’-GCCTCCTTTCCACCTTTTGT-3’	5’-GATTCAAACACAGCATAGATGGAC-3’

primer squences for real-time PCR

### Immunohistochemistry

Soleus and plantaris muscles were directly placed in formalin solution (Sigma, St. Louis, MO). Cross-sections were cut from the mid-belly region of each muscle. Formalin-fixed paraffin-embedded sections (5μm) were deparaffinized, hydrated and antigen retrieval was performed by xylene. The tissue was permeabilized with 0.02 % Triton X-100 in PBS (PBST) for 15 min and blocking with 5 % BSA in PBST for 30 min. Next, the slides were washed once with PBS, after which they were probed with FATP1, FAT/CD36 polyclonal rabbit antibody and FABPpm monoclonal mouse antibody (Abcam, Cambridge, UK) at a dilution of 1:200 overnight at 4℃ in 5 % BSA in PBS. The slides were then washed thrice for 5 min each in 0.05 % Tween 20 in PBS, after which they were incubated with Alexa 488-conjugated goat anti-rabbit or Alexa 568-conjugated goat anti-mouse IgG secondary antibody (Invitrogen Life Technologies, Carlsbad, CA) diluted 1:200 in PBS that contained 5 % BSA for 20 min at room temperature. Next, the slides were washed thrice with 0.05 % Tween 20 in PBS, after which they were mounted with mounting media. The slides were then viewed and photographed using a Nikon Imaging System (Nikion, Tokyo, JAPAN).

### Data Analysis

Results were presented as mean ± standard error of the mean of observations. Statistical analysis was performed using SPSS 22.0. Between-group differences (exercise groups vs. non-exercise groups and treatment groups vs. non-treatment groups) were analysed using two-way analysis of variance (ANOVA). Comparisons between multiple treatment groups were performed using Turkey’s post-hoc tests. In all analyzes, the values were considered significantly different at values of p<0.05.

## RESULTS

### Effects of PR and exercise on fatty acid transporters mRNA expression in soleus muscles

**Figure 2.**
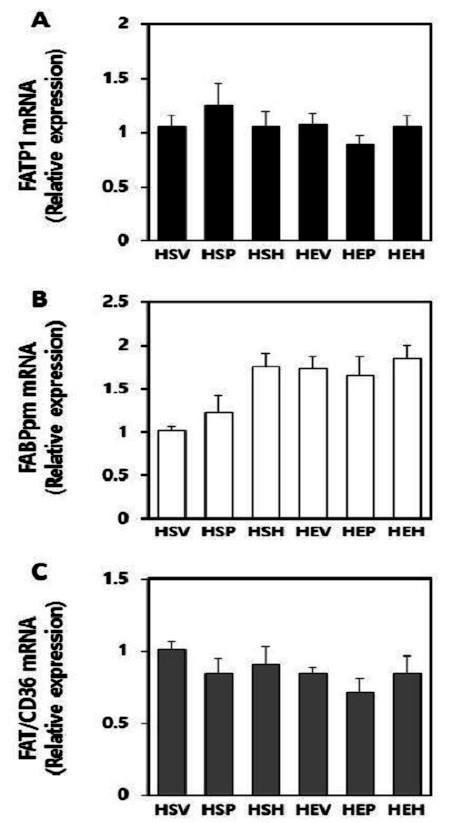
Target mRNA levels determined by real-time qPCR in OVX rats soleus muscle. (A) FATP1, (B) FABPpm, and (C) FAT/CD36. Target mRNA values are normalized to the GAPDH mRNA level for each samples. Samples were analyzed in duplicate in parallel with GAPDH. Values are means ± SE from nine independent experiments.

To determine the effects of PR, 17β-estradiol and exercise on fatty acid transporters gene expression in OVX rat soleus muscles, fatty acid transporters mRNA level was analyzed by using real-time qPCR (Fig. 2). There was no significant alteration in FATP1 mRNA level (Fig. 2A). Whereas, either exercise (p=0.003) or treatment (p=0.02) had main effect on up-regulation of FABPpm gene expression. Post-hoc test results showed that FABPpm mRNA expression was significantly increased in 17β-estradiol treatment groups, as compared with vehicle groups (p=0.025). However, exercise combined with PR or hormone had no additive effects on FABPpm gene expression (Fig. 2B). As shown in Fig. 2C, FAT/CD36 mRNA expression was not significantly altered in this study.

### Effects of PR and exercise on fatty acid transporters mRNA expression in plantaris muscles

**Figure 3.**
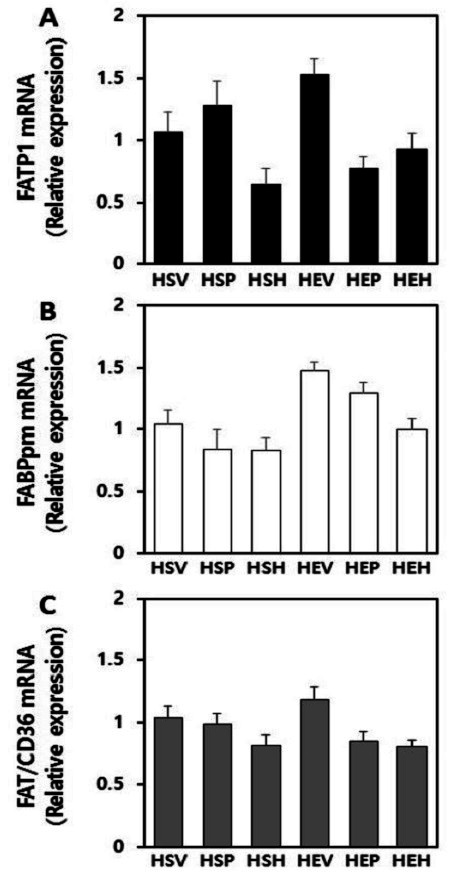
Target mRNA levels determined by real-time qPCR in OVX rats plantaris muscle. (A) FATP1, (B) FABPpm, and (C) FAT/CD36. Target mRNA values are normalized to the GAPDH mRNA level for each sample. Samples were analyzed in duplicate in parallel with GAPDH. Values are means ± SE from nine independent experiments.

Real-time qPCR analysis revealed that fatty acid transporters gene expression varied in response to treatment with exercise, PR or 17β-estradiol in OVX rats plantaris muscle (Fig. 3). Exercise does not alter FATP1 gene level in plantaris muscles. Unexpectedly, treatment had main effect on down-regulation of FATP1 mRNA level (p=0.005). Post-hoc test results showed that FATP1 mRNA expression was significantly decreased in 17β-estradiol intake groups when compared with vehicle groups (p=0.004). Furthermore, exercise combined with supplement had interaction effects on FATP1 gene suppression (p=0.004) (Fig. 3A). As shown in Fig. 3B, exercise had main effect on up-regulation of FABPpm mRNA level (p=0.000). whereas treatment had main effect on down-regulation of FABPpm mRNA level (p=0.005). Post-hoc test results showed that especially in 17β-estradiol intake groups, FABPpm gene level was significantly decreased, as compared with vehicle groups (p=0.003). Because of these contradictory results, exercise combined with treatments had no additive effect on FABPpm mRNA expression in plantaris muscle (Fig. 3B). On the contrary, FAT/CD36 mRNA expression showed no significant alteration (Fig. 3C).

### Effects of PR and exercise on fatty acid transporters protein expression in soleus muscles

**Figure 4.**
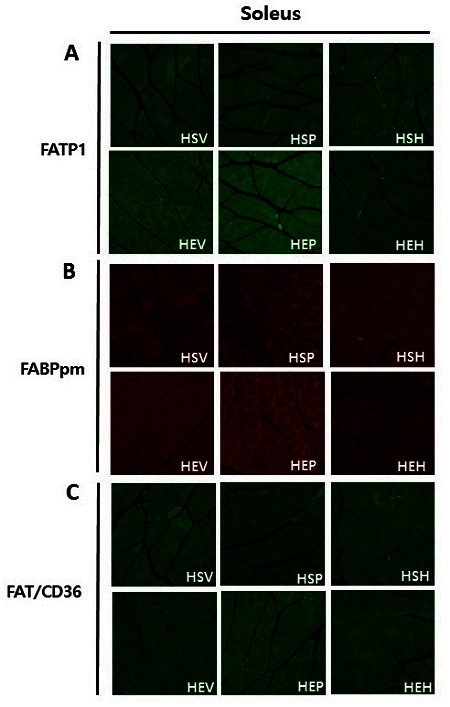
Immunohistochemistry image of fatty acid transporters stained with skeletal muscle cross-sections of OVX rat soleus muscle. FATP1 (green, A), FABPpm (red, B) and FAT/CD36 (green, C) was detected by immunofluorescence.

Immunohistochemistry was conducted to identify the effects of PR and exercise on fatty acid transporters protein expression in OVX rat soleus muscles. Immunohistochemistry (IHC) results showed that in vehicle and PR intake groups, FATP1 proteins were highly expressed and translocated from an intracellular depot to the plasma membrane in exercise groups, as compared with sedentary groups (green, Fig. 4A). In contrast, in hormone intake groups, FATP1 proteins were expressed less in exercise groups, as compared with the sedentary group. In all groups (vehicle, PR, and estradiol intake groups), FABPpm proteins expression was higher and localized at plasma membrane dominantly in exercise groups, as compared with sedentary groups (red, Fig. 4B). Although there was a subtle difference between all groups, FAT/CD36 proteins were more highly expressed in exercise groups than sedentary groups (green, Fig. 4C). These data indicates that in exercise groups compared with sedentary groups, PR and exercise lead fatty acid transporters proteins expression was increased and translocated from cytosol to plasma membrane in OVX rat soleus muscles.

### Effects of PR and exercise on fatty acid transporters protein expression in plantaris muscles

**Figure 5.**
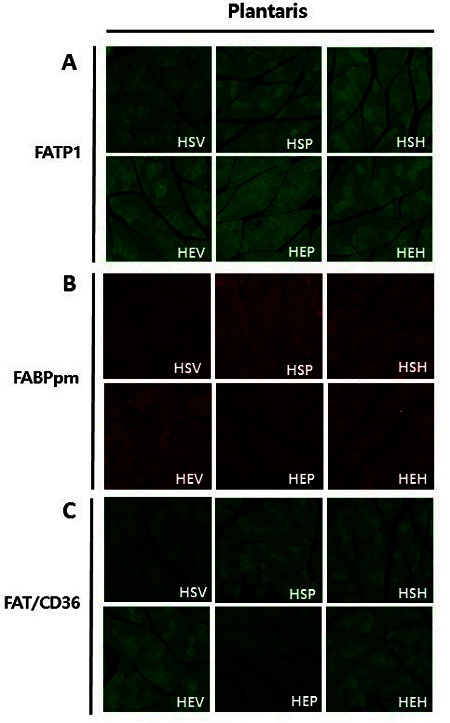
Immunohistochemistry image of fatty acid transporters stained with skeletal muscle cross-sections of OVX rat plantaris muscle. FATP1 (green, A), FABPpm (red, B) and FAT/CD36 (green, C) was detected by immunofluorescence.

To determine whether PR and exercise-induced increase in fatty acid transporters protein localization was determined by IHC in OVX rats plantaris muscles. In vehicle, PR, and estradiol intake groups, FATP1 proteins were translocated from intracellular depot to plasma membrane and highly expressed in exercise groups than sedentary groups (green, Fig. 5A). Exercise groups represented high FABPpm proteins expression and translocation from intracellular depot to plasma membrane, as compared with sedentary groups (red, Fig. 5B). In addition, the results indicated that FAT/CD36 proteins were highly expressed and localized at plasma membrane in exercise group in comparison with the other groups (green, Fig. 5C). Thus, in exercise groups in comparison with sedentary groups, PR and exercise induce fatty acid transporters expression and translocation to the plasma membrane in OVX rats plantaris muscles.

## DISCUSSION

Lipid metabolic problems in postmenopausal women such as insulin resistance, dyslipidemia, and cardiovascular disease may result from impaired balances between fatty acid uptake and oxidation^8^^,^ ^22^. ERT is a widely used treatment for estrogen deficiency but many studies have reported its side effects. Therefore, either PR or exercise has been receiving much attention as an alternative of ERT and show a reducing effect on fat mass in postmenopausal women^23^. However, few studies have shown the effects of exercise, PR, or exercise combined with PR on fat metabolism through the FATPs, which play important roles in mediating lipid metabolism by transporting fatty acid, in postmenopausal model^24^. Therefore, we examined the effects of exercise, PR, and exercise combined with PR on FATP1, FABPpm and FAT/CD36, which play important roles in maintaining the balance between lipid uptake and oxidation in obese induced OVX rat skeletal muscles.

First, in this study, though exercise had no main effect on FATP1 mRNA expression, in the exercise groups, FATP1 proteins showed increased translocation from intracellular depot to plasma membrane in soleus and plantaris. Thus, FATP1 expression is induced by exercise. However, Dyck et al.^25^ likewise showed that FATP1 mRNA expression was not altered in rat soleus and red gastrocnemius muscles after 8 weeks of endurance training. They reported that their exercise intensity was not sufficient to increase FATP1 mRNA expression, hence, it is likely that, the exercise intensity in our study might be considered insufficient to alter the FATP1 mRNA level. Another surprising finding was that FATP1 mRNA level was significantly decreased in 17β-estradiol treatment groups, as compared with vehicle groups in plantaris muscles. However, no previous study or scientific base could explain our results.

Although exercise or exercise combined with PR had no main effects on FATP1 gene level, plasma membrane localization of FATP1 protein was high. PR and exercise have additive effects on FATP1 protein localization to plasma membrane by pre-translational mechanisms. Translocation of FATP1 proteins from intracellular depot to plasma membrane is related with capacity of fatty acid uptake and oxidation in mammals^26^. Therefore, our result indicates better lipid metabolism in exercise and exercise combined with PR groups, as compared with other groups.

Second, an important feature of this study is that FABPpm gene expression was significantly higher in exercise groups than non-exercise groups in soleus and plantaris muscles. Moreover, FABPpm protein localization of plasma membrane was increased not only in the exercise group but also in the exercise combined with PR group in both muscles. These results are in line with previous studies that aerobic exercise induces up-regulation of FABPpm mRNA, and protein translocation to plasma membrane. It also implies that the capacities of fatty acid uptake and oxidation in skeletal muscles are increased^27^^,^ ^28^. Furthermore, exercise and PR had beneficial effects on improving impaired lipid metabolism in OVX rat skeletal muscles. Another important feature of this study is that the soleus muscles showed a greater range of increased FABPpm mRNA expression than plantaris muscles. Bonen et al.^29^ reported that the higher FABPpm was contained in red muscles than in white muscles and also the palmitate transport capacity was higher in red muscle in comparison with white. This indicates that red muscles, which are oxidative muscles, have greater number of fatty acid transporters and fatty acid transport capacity. Taken together, our result indicates that soleus muscles have the higher capacity of fatty acid utilization by muscle specific fatty acid transporter, FABPpm.

Third, the results of this study showed that FAT/CD36 mRNA expression was not altered in exercise groups, as compared with non-exercise groups in rat skeletal muscles. Whether FAT/CD36 expression is higher or not is a controversial issue. According to Kiens et al.^30^, there were no significant effects of training status on the mRNA and protein levels of FAT/CD36 in human skeletal muscles. However, FAT/CD36 mRNA expression was significantly increased after 90 min of acute bicycle exercise. In the present study, FAT/CD36 mRNA expression was not altered by aerobic exercise for 8 weeks. Taken together, exercise might induce the immediate increase of FAT/CD36 mRNA expression after a single bout exercise, and FAT/CD36 content in skeletal muscle may not be an adaptation of low-intensity aerobic exercise. Nevertheless, aerobic exercise induces translocation of FAT/CD36 protein from cytosol to plasma membrane that is required to increase the fatty acid transport into the mitochondria and support ATP production^21^. In this study, FAT/CD36 proteins were more highly expressed and localized at plasma membrane in exercise groups, as compared with the non-exercise groups in both muscles. These results supported that FAT/CD36 expression may have been regulated by pre-translational mechanisms in OVX rat skeletal muscles.

In summary, the results of this study showed that exercise had main effect on up-regulation of FABPpm mRNA alone. This result implies that aerobic exercise induces increased fatty acid uptake in OVX rat skeletal muscles through up-regulation of FABPpm mRNA expression, which are known as muscle-specific fatty acid transporter. These results also represent that there were no additive effects of exercise combined with other treatments on any FATPs mRNA expression. However, translocation of FATPs proteins to plasma membrane was higher in PR groups and exercise combined with PR groups, as well as exercise only group. These results showed that FATPs protein localization might be regulated by pre-translational mechanisms in OVX rat skeletal muscles. In this study, exercise intensities or PR concentrations may not adequately regulate FATP1 and FAT/CD 36 mRNA expression. The mechanisms of fatty acid transporters by exercise and PR require future studies with various intensities or periods. Moreover, further studies are required to demonstrate the roles of fatty acid transporters in mitochondria and the effects of exercise combined with PR on mitochondria biogenesis, which is an essential lipid metabolism of skeletal muscles. Further studies are required on the effects of PR and exercise on lipid metabolism in skeletal muscles of postmenopausal women.
